# Purification, Characterization, and Potential of Saline Waste Water Remediation of a Polyextremophilic **α**-Amylase from an Obligate Halophilic *Aspergillus gracilis*

**DOI:** 10.1155/2014/106937

**Published:** 2014-05-14

**Authors:** Imran Ali, Ali Akbar, Benjawan Yanwisetpakdee, Sehanat Prasongsuk, Pongtharin Lotrakul, Hunsa Punnapayak

**Affiliations:** ^1^Plant Biomass Utilization Research Unit, Department of Botany, Faculty of Science, Chulalongkorn University, Bangkok 10330, Thailand; ^2^Institute of Biochemistry, University of Balochistan, Quetta 87300, Pakistan; ^3^Food Engineering and Bioprocess Technology, School of Environment, Resources and Development, Asian Institute of Technology, Klong Luang, Pathumthani 12120, Thailand

## Abstract

An obligate halophilic *Aspergillus gracilis* which was isolated from a hypersaline man-made saltern from Thailand was screened for its potential of producing extracellular **α**-amylase in the previous studies. In this study the **α**-amylase was extracted and purified by the help of column chromatography using Sephadex G-100 column. Presence of amylase was verified by SDS-PAGE analysis, showing a single band of approximately 35 kDa. The specific activity of the enzyme was found to be 131.02 U/mg. The Lineweaver-Burk plot showed the *V*_max_ and *K*_*m*_ values of 8.36 U/mg and 6.33 mg/mL, respectively. The enzyme was found to have the best activity at 5 pH, 60°C, and 30% of NaCl concentration, showing its polyextremophilic nature. The use of various additives did not show much variation in the activity of enzyme, showing its resilience against inhibitors. The enzyme, when tested for its use for synthetic waste water remediation by comparing its activity with commercial amylase in different salt concentrations showed that the **α**-amylase from *A. gracilis* was having better performance at increasing salt concentrations than the commercial one. This shows its potential to be applied in saline waste water and other low water activity effluents for bioremediation.

## 1. Introduction

Extremophiles are able to reproduce and grow at extremes of pH, temperature, salinity, and so forth. Halophiles are those extremophiles which are able to withstand extremes of salinity [1]. Halophilic fungi can be defined as those which are frequently found in hypersaline habitats at NaCl concentrations above 1.7 M and they are able to grow* in vitro* at 3 M salt concentration [2, 3]. Halophilic fungi which are unable to grow without the presence of required NaCl concentrations are called as obligate halophilic fungi [1].

Extremophiles have long been studies for their metabolites which are capable of working at extreme available conditions [4]. Currently there are several fermentation processes in which halophiles and their metabolites are used [5]. There are reports of using halophilic metabolites as food additives, biosurfactants, biorhodopsins, and biocompatible solutes [3, 6]. The ability of extremophiles to produce hydrolytic extremozymes has been much studied for its possible applications in industries [7, 8]. Mostly halophilic hydrolases such as amylases, cellulases, lipases, xylanases, and proteases have been reported from halophilic bacteria [9]. Except for few preliminary studies there have not been many investigations on the extremozymes from halophilic fungi, particularly obligate halophilic fungi [3, 10].

Amylases are potent industrial enzymes used in textile, laundry, pharmaceutical, and food industries [11, 12]. The *α*-amylase (EC 3.2.1.1) is an important class of amylases which constitutes approximately 25% of its share in total enzyme market [13].

*Aspergillus gracilis* TISTR 3638 was isolated from a man-made solar saltern, present in Phetchaburi province of Thailand. The isolate was morphologically and molecularly identified. Characterization studies of fungus revealed the obligate halophilic nature of the fungi [1]. The strain was then deposited to the culture collection center of Thailand Institute of Scientific and Technological Research (TISTR). The fungus was found to have extracellular amylase (see Supplementary Figure 1 in the Supplementary Material available online at http://dx.doi.org/10.1155/2014/106937), when its biotechnological potentials were investigated [3].

It was assumed from previous studies on other halophilic microbes that the amylase from* A. gracilis* may have polyextremophilic nature which can make it applicable in many industrial processes. So, in quest of that, the purification and characterization of *α*-amylase were performed. The purified amylase was tested for its potential in the use of waste water management. To the best of our knowledge this is the first report of use of amylase for waste water remediation from any halophilic fungi, especially from obligate halophilic fungi.

## 2. Materials and Methods

### 2.1. Growth Conditions for Enzyme Production

Fresh culture of* Aspergillus gracilis* was grown on the potato dextrose agar (PDA) supplemented with 1% (w/v) soluble starch and 10% (w/v) of NaCl concentration. Five mm of two discs obtained by cock borer was inoculated in 100 mL of production medium in 150 mL Erlenmeyer flask.* A. gracilis* was grown in the production medium at room temperature (25 ± 2°C) at 150 rpm for 14 days. The medium for amylase production was made by following Hernàndez et al. [14] with required modifications.* A. gracilis *was found growing best at 10% salt concentration in our previous finding [1], so the medium was supplemented with 10% NaCl (w/v). The medium was composed of 8.0 g/L CaCO_3_, 0.15 g/L FeSO_4_·7H_2_O, 3.5 g/L KH_2_PO_4_, 0.10 g/L MgSO_4_·7H_2_O, 3.0 g/L mycological peptone, 100 g/L NaCl, 6.6 g/L (NH_4_)_2_SO_4_, and 10 g/L soluble starch.

### 2.2. Amylase Purification

The methodologies adopted by previous researchers [15, 16] for the purification of amylase were followed. Supernatant from the growth media was obtained by centrifuging the broth at 13,000 ×g at 4°C for 10 min. The enzyme was precipitated by the addition of solid ammonium sulfate bringing the filtrate to 90% saturation. The mixture was incubated overnight at 4°C. The precipitate obtained was then centrifuged for 30 min at 12,000 ×g and dissolved in 100 mM Tris-HCl buffer (pH 6). The enzyme was then dialyzed for 48 h using same buffer. The precipitate obtained was then applied for gel filtration by Sephadex G-100 column (2.6 cm–150 cm), which was preequilibrated with 25 mM Tris-HCl buffer (pH 6, containing 0.5% Triton X-100). Enzyme fractions of 5 mL were eluted by using same buffer at a flow rate of 30 mL/h.

The purity and molecular weight of the purified amylase were determined by sodium dodecyl sulphate polyacrylamide gel electrophoresis (SDS-PAGE). The methodology explained by Hmidet et al. [16] was followed by using 5% stacking and 15% separating gels. The purified enzyme was mixed at 1 : 5 ratio (v/v) with distilled water having 10 mM Tris-HCl (pH 6), SDS 2.5%, b-mercaptoethanol 5%, glycerol 10%, and bromophenol blue 0.002%. Before electrophoresis the sample was heated for 5 min at 100°C. Gels were stained with 0.25% Coomassie Brilliant Blue R250 in 45% ethanol-10% acetic acid and destained with 5% ethanol-7.5% acetic acid. Molecular marker kit (Bio-Rad) was used for the reference of molecular weights of protein.

### 2.3. Amylase Assay

Activity of amylase was determined by dinitrosalicylic acid (DNS) method of reducing sugar explained by Miller [17]. 0.1 mL amylase was added with 0.5 mL of 0.1 M acetate buffer having 1% (w/v) soluble starch. The reaction mixture was incubated for 10 min at 40°C. The reaction was stopped by 3 mL of 3,5-dinitrosalicylic acid and the mixture was heated at 100°C for 5 min in boiling water bath. 10 mL of water was added and the absorbance was determined at 540 nm. One unit of enzyme activity (U) was defined as the amount of enzyme that produced 1 *μ*mol of glucose in 1 min.

### 2.4. Protein Estimation

The amount of protein was determined by following methodology adopted by Lowry et al. [18]. Bovine serum albumin (BSA) was used as the standard.

### 2.5. Characterization of Amylase

The amylase was characterized in various pH, temperatures, and NaCl concentrations. The results were explained in percentage relative activity.

The pH range of 3–8 at constant temperature was provided for the determination of effect of pH on enzyme activity. For acidic range of pH 0.1 M acetate buffer was used, while for neutral and alkaline ranges of pH 0.1 M phosphate buffer was utilized. The sample mixture was incubated at 30–90°C at constant pH for the determination of effect of temperatures on enzyme activity. The effect of salinity on the enzyme activity was determined by supplementing the sample mixture at 0–40% NaCl concentration (w/v) at constant pH and temperature.

The effects of various activators and deactivators on the enzyme activity were determined by adding 2 mM b-mercaptoethanol, FeCl_2_, ethylene diamine tetra-acetic acid (EDTA), CaCl_2_, BaCl_2_, MgCl_2_, HgCl_2_, and ZnCl_2_. The effect of additives was compared with the control having no additive. The results are explained in percentage relative activity.

### 2.6. Determination of Kinetic Parameters

The kinetic parameters were estimated by incubating the enzyme at various concentrations of soluble starch. The optimum conditions found in the characterization studies were used in kinetic studies. Lineweaver-Burk plot was used for finding the* V*_max_ and* K*_*m*_ values.

### 2.7. Waste Water Treatment

The potential of amylase from* A. gracilis* was determined for waste water remediation. The performance was checked by comparing the efficiency with commercial grade amylase. Synthetic waste water was made following the composition used by Kapdan and Erten [19] with few modifications. The medium was composed of soluble starch 10 g/L, NH_4_Cl 1 g/L, KH_2_PO_4_ 0.3 g/L, MgCl·6H_2_O 2 g/L, CaCl_2_·2H_2_O 0.2 g/L, C_2_H_3_NaO_2_·3H_2_O 1 g/L, and trace element solution 1 mL/L. The trace element solution was made by MgSO_4_·7H_2_O 3 g/L, MnSO_4_·2H_2_O 0.5 g/L, FeSO_4_·7H_2_O 0.1 g/L, CaCl_2_·2H_2_O 0.1 g/L, ZnSO_4_·7H_2_O 0.180 g/L, CuSO_4_·5H_2_O 0.01 g/L, H_3_BO_3_ 0.01 g/L, Na_2_MoO_4_·2H_2_O 0.01 g/L, and NiCl_2_·6H_2_O 0.25 g/L.

The sample mixtures were made by the addition of 10% of amylase from* A. gracilis* and commercial amylase. The sample mixtures were supplemented with 0–25% of NaCl concentrations and incubated for 1 hour at constant pH and temperature. Blank was used as negative control having no enzyme. The change in dissolved oxygen (DO) was monitored by DO meter (DO-5519 Lutron, Taiwan). The results are explained in percentage relative efficiency, where the increase in DO by the amylase from* A. gracilis* was considered as control and its DO values were taken as 100%.

### 2.8. Statistical Analysis

Experiments were performed with required controls. Data is presented as mean ± standard deviation (SD) of triplicate readings. A value of  *P* < 0.05 was considered significant.

## 3. Results

### 3.1. Purification of Amylase

**Figure 1:**
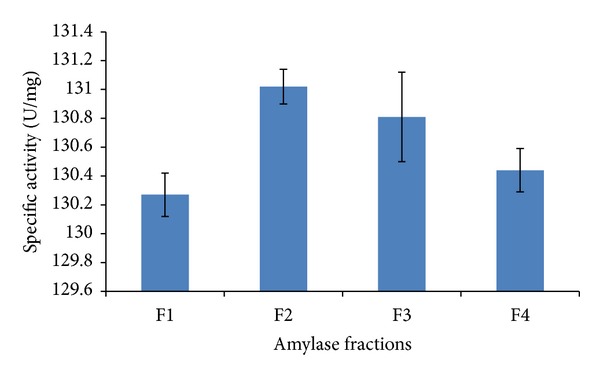
Fractions of *α*-amylase obtained from* A. gracilis* TISTR 3638, produced at flow rate of 30 mL/h by column chromatography.

**Figure 2:**
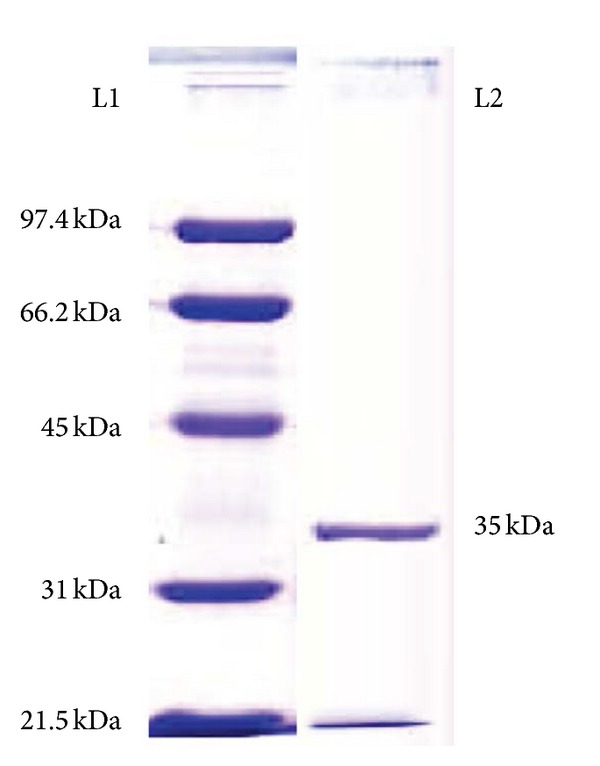
SDS-PAGE analysis of the purified *α*-amylase from* A. gracilis* TISTR 3638. L1 represents lane 1, which is the molecular mass ladder. L2 represents lane 2, which is the purified *α*-amylase lane, showing single band at approximately 35 kDa.

The purity and molecular weight of *α*-amylase was determined from the F2 fraction showing highest amylase activity (Figure 1). The purified amylase was found to have a single band at approximately 35 kDa (Figure 2). The specific activity of the purified amylase from* A. gracilis* was found as 131.02 U/mg. Approximately 6 folds of purification were found by purification with 47% yield (Table 1).

### 3.2. Characterization of Amylase

**Figure 3:**
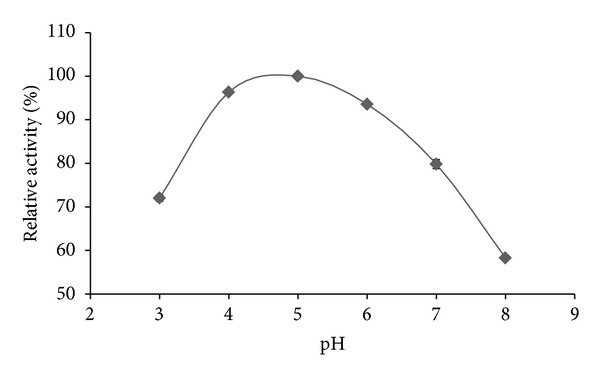
Effect of pH on the purified *α*-amylase activity from* A. gracilis* TISTR 3638 at constant temperature. The results are expressed in percentage relative activity.

The effect of different pH ranges on the enzyme activity showed that amylase was having high activity at acidic to neutral pH ranges (Figure 3). Increase in pH was found to decrease the enzyme activity. The optimum enzyme activity was found at 5 pH.

**Figure 4:**
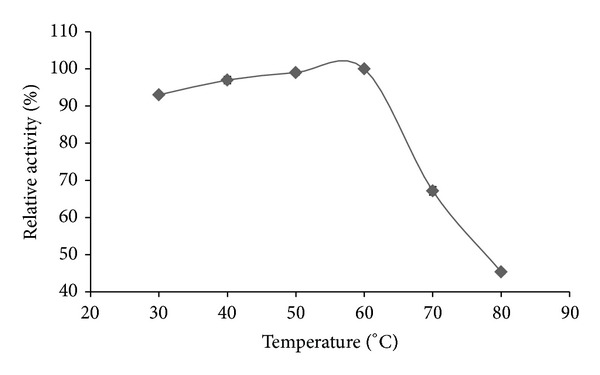
Effect of temperature on the purified *α*-amylase activity from* A. gracilis* TISTR 3638 at constant pH. The results are expressed in percentage relative activity.

The effect of different temperatures on enzyme activity showed that amylase was able to work better from moderate to high temperatures (Figure 4). Steady increase in enzyme activity was observed from 30 to 60°C. A sharp decrease in enzyme activity was found at temperatures over 60°C.

**Figure 5:**
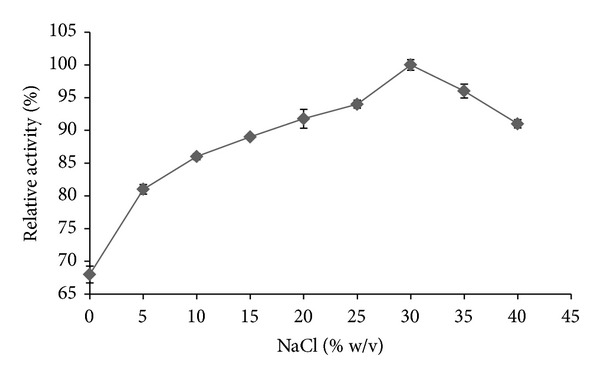
Effect of NaCl concentrations (% w/v) on the purified *α*-amylase activity from *A. gracilis* TISTR 3638 at constant pH and temperature. The results are expressed in percentage relative activity.

The effect of various salinity concentrations on amylase activity revealed the halophilic nature of the enzyme (Figure 5). A gradual increase in enzyme activity was observed at increasing NaCl concentrations. The optimum enzyme activity was observed at 30% salt concentration. The enzyme was able to show over 30% more efficiency at very high salt concentration of 30% as compared to 0% salt concentration. The decrease in enzyme activity was observed from salinity above 30% of NaCl concentration but still more than 90% of enzyme activity was retained at 40% of NaCl concentration which is above the saturation point of saline solutions.

The effect of various additives on the enzyme exhibited its resilience to enzyme inhibition (Table 2). None of the inhibitors was able to decrease lower than 90% of enzyme activity. However the activators were also not able to influence the enzyme activity as well. Over all BaCl_2_, MgCl_2_, HgCl_2_, and CaCl_2_ were able to increase the enzyme activity, while FeCl_2_, b-mercaptoethanol, ZnCl_2_, and EDTA were able to decrease the enzyme activity.

### 3.3. Enzyme Kinetics

**Figure 6:**
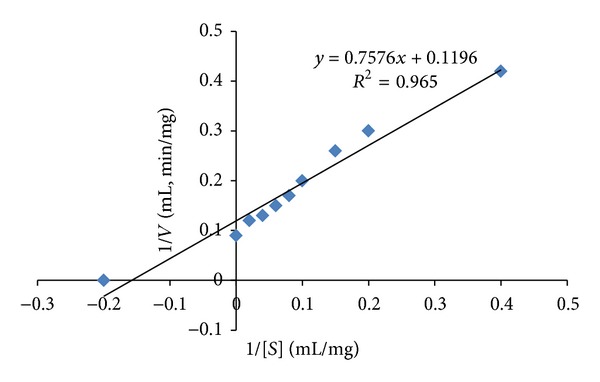
Lineweaver-Burk plot for the determination of* V*_max_ and* K*_*m*_ values of the purified *α*-amylase from* A. gracilis* TISTR 3638, at optimum conditions, in the presence of different concentrations of soluble starch.

The enzyme kinetics was determined by Lineweaver-Burk plot (Figure 6). The* V*_max_ and* K*_*m*_ values of amylase from* A. gracilis* were found to be 8.36 U/mg and 6.33 mg/mL, respectively.

### 3.4. Waste Water Treatment

**Figure 7:**
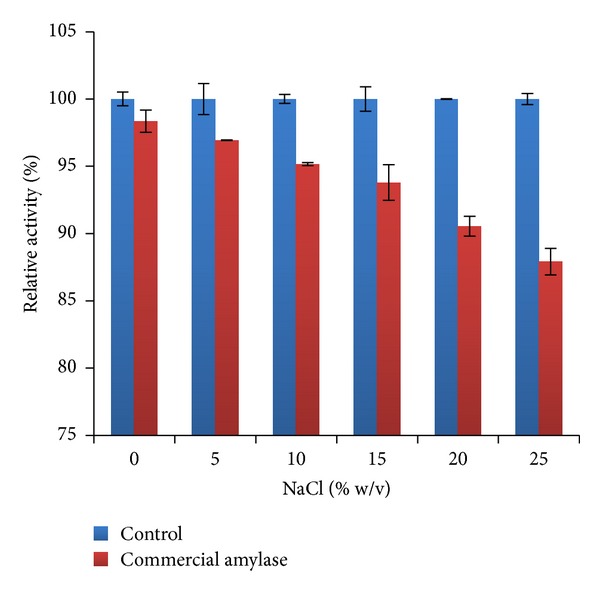
Comparative analysis of waste water remediation between control (*α*-amylase from *A. gracilis* TISTR 3638) and commercial grade amylase at different salinity levels.

Remediation of synthetic waste water was monitored by increase in the DO. In comparison to the performance of amylase from* A. gracilis* (control) the efficiency of commercial amylase was found to be decreased by the increase in NaCl concentrations (Figure 7).

## 4. Discussion

There is a constant search by researchers for finding suitable fungal strains for the amylase production [20]. Mesophilic fungi have been mostly reported for the production of amylases [21]. The amylase production has been dominated by* Aspergillus* and* Penicillium *genus [22]. In industrial operations the amylases from fungi are preferred due to their acceptable characteristics such as in food and pharmaceutical industries [23]. The species from* Aspergillus genus*, such as* Aspergillus oryzae *and* Aspergillus niger* are commonly used for the production of amylases in industries [24]. Fungi were not designated as halophilic microorganisms before year 2000 [2]. Therefore there are not many reports of amylase from halophilic fungi. To the best of our knowledge this is the first attempt of using amylase for remediation of waste water from halophilic fungi, especially obligate halophilic fungi.

A single band obtained in purification step suggests that there is no further need of any purification of this enzyme. The molecular masses from halophilic fungi have been mostly reported in the range of 50–75 kDa [9]. The *α*-amylase obtained from* A. gracilis *TISTR 3638 was found to be smaller in this study with an approximate mass of 35 kDa (Figure 2). The specific activity of amylase in this study was found in the moderate ranges as compared to previous reports [15]. The* K*_m_ value of the amylases has been mostly reported from 0.35 to 11.66 mg/mL [25], which corresponds to our report.

The optimum pH value of amylase from this study is found in the acidic range, which is slightly different to the growth characterization results of* A. gracilis* 3638, as it was found growing best at 7 pH [1]. Similarly, it has also been found different to the habitat conditions of hypersaline environment, as mostly the hypersaline environments are found to have pH values from neutral to slightly alkaline [5]. The optimum pH value of 5 has been considered as acidophilic by researchers [26, 27]. Acidophilic amylases can work better in acidic waste water [28] and they can work better at extremes of acidic pH in the stomach [27].

Most of the enzymes are reported being unable to work at temperatures more than 50–60°C [29]. The optimum activity of amylase in this study has been found at 60°C, which is near to be the standard thermophilic category of amylases, which is 70–80°C [30, 31]. Thermophilic amylases are mostly used in the starch and baking industries [29].

Obligate halophilic fungi are unable to survive in the absence of required salt concentration. The minimal required salt concentration for* A. gracilis* TISTR 3638 has been found at 5% NaCl (w/v) [1]. This characteristic provided a hypothesis for this study that amylase must be requiring salt concentration for better activity. Salt concentration of 10% (w/v) has been mostly found best for the production of amylases from halophilic microorganisms [13, 32]. Consider that 10% of salt was supplemented in the production media of amylases, which signifies the halophilic characteristic of enzyme production. Amongst halophiles, the Archaea and bacteria are considered most extreme halophilic microbes and their metabolites are also considered extremely halophilic.. Amylase in this study, being able to perform best at 30% of NaCl concentration, has been found more extremely halophilic as compared to many reports on amylases from halophilic archaea and bacteria [8, 13, 33]. The halophilic extremity of amylases in this study adds the novelty to this enzyme. This finding also provides an insight into the fact that metabolites from halophilic fungi have been much neglected in biotechnology, although they have the ability to work better at low water activity or high salt concentrations' processes in industries.

The halophilic amylases from microbial sources have often been reported to work better at more than one extremity of available conditions, thus showing the polyextremophilic behavior [8, 13]. Amylase in this study has been found being acidophilic, thermophilic, and halophilic: making it fall into the category of polyextremophilic enzymes. However, the characteristic of amylase from* A. gracilis* TISTR 3638 has been found different from the typical behavior of extremozymes obtained from halophilic microorganisms, as most of them are found to be haloalkaliphilic [9]. Enzymes having ability to work at extremes of available conditions are found more potent for application in industrial processes [34]. The polyextremophilic behavior shown by amylase in this study makes it available to work at low pH, high temperatures, low water activity, and high salt concentration processes in industries.

Climate changes and continuously changing world are requiring new demands from biotechnology. There are several places in the world where people have to use underground untreated water for domestic purposes, which decreases the performance of many detergents [35–37]. The use of amylase from this study as an additive in the laundry detergents can solve this problem and can also reduce the consumption of water. Depletion of fossil fuels requires the use of biofuels as alternatives. Halophilic enzymes are also being reported to work better than normal enzymes in the biofuel production processes [38].

Similarly, the increased demands of food, clothes, and other utilities have increased the demand of production from industries, which is ultimately causing increase in waste water production [39]. Addition of any pollutant in the waste water decreases the DO in the water. Several food, pharmaceutical, and textile industry effluents are rich in starch, fiber, and other organic pollutants which are very harmful for environment [40]. In addition to that, there are various industries producing saline and acidic waste water such as fertilizer industries, fish sauce production units, and phenol production industries, which makes the remediation process very difficult [28, 41, 42]. Considering these problems we practically checked the performance of amylase from* A. gracilis* TISTR 3638 with commercial grade amylase. Both amylases were able to increase the DO of the synthetic waste water. The results show that increasing salinity increased the performance of amylase from* A. gracilis* TISTR 3638, while it decreased the performance of commercial amylase. The use of halophilic enzymes has already been reported for the bioremediation of saline areas as well as for treatment of saline waste water [43, 44]. As our enzyme has been found more salt loving than reported halophilic amylases, it provides the possibility of using it in saline and low water activity waste management. In addition to that the resilience of our reported amylase to inhibitors also makes it more potential candidate in waste water remediation processes as industrial effluents are often found to be contaminated with metallic ions as well.

## 5. Conclusions

The metabolites available from halophilic and obligate halophilic fungi have been much investigated. The polyextremophilic behavior of *α*-amylase obtained from obligate halophilic* A. gracilis* TISTR 3638 provides the options of using it in industrial operations occurring in mild acidic and temperature conditions and at low water activity. The comparison of *α*-amylase from this study with the commercial grade amylase for waste water remediation has shown that the amylase from* A. gracilis* TISTR 3638 can be applied for saline waste water remediation. There is still need for more in-depth studies to understand the biochemical behavior of *α*-amylase from* A. gracilis* TISTR 3638.

## Supplementary Material

Supplementary Figure 1: Enzyme plate screening of **α**-amylase from *A. gracilis* TISTR 3638 on Potato Dextrose Agar (PDA) supplemented with 1% soluble starch (w/v). The fungal colony was grown for one week. The clear zone is highlighted by the help of iodine solution.

## Figures and Tables

**Table 1:** Purification properties of *α*-amylase from *A. gracilis* TISTR 3638.

Properties	Cell free supernatant	(NH_4_)_2_SO_4_ precipitation	Gel purification
Total protein (mg)	215.54 ± 1.21	634.33 ± 0.85	183.51 ± 0.82
Total activity (U)	45718.27 ± 0.93	36207.55 ± 0.07	24043.48 ± 1.15
Specific activity (U/mg)	21.19 ± 0.47	57.08 ± 1.02	131.02 ± 0.43
Yield (%)	100	73	47
Purification fold	1.0	2.69	6.18

**Table 2:** Effect of various additives on the *α*-amylase from *A. gracilis* TISTR 3638.

Additives (2 mM)	Relative activity (%)
None	100
BaCl_2_	101.05 ± 1.5
CaCl_2_	101.47 ± 0.1
FeCl_2_	89.42 ± 1.0
HgCl_2_	100.24 ± 1.5
MgCl_2_	100.33 ± 0.3
ZnCl_2_	90.38 ± 1.5
b-Mercaptoethanol	98.01 ± 0.5
EDTA	90.41 ± 1.0
