# Supplementary material for: Purification, Characterization, and Potential of Saline Waste Water Remediation of a Polyextremophilic α-Amylase from an Obligate Halophilic Aspergillus gracilis
Source: Biomed Res Int. 2014 May 14;2014:106937. doi: 10.1155/2014/106937 (PMC4053144; doi:10.1155/2014/106937)
Supplement: Supplementary file 1 — Supplementary Figure 1: Enzyme plate screening of α-amylase from A. gracilis TISTR 3638 on Potato Dextrose Agar (PDA) supplemented with 1% soluble starch (w/v). The fungal colony was grown for one week. The clear zone is highlighted by the help of iodine solution. [file 106937.f1.docx]

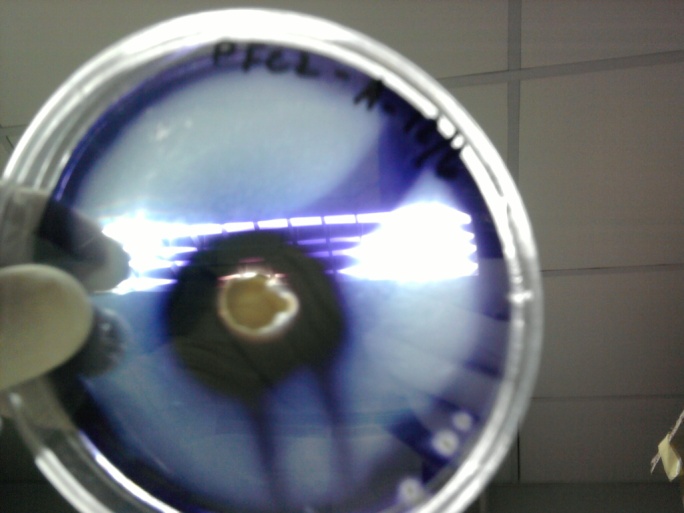


Supplementary Figure 1: Enzyme plate screening of α-amylase from *A. gracilis* TISTR 3638 on PDA supplemented with 1% soluble starch (w/v). The clear zone is highlighted by the help of iodine solution.
